# Beyond associations: Navigating the safety of non-steroidal anti-inflammatory drugs (NSAIDs) in early pregnancy

**DOI:** 10.1371/journal.pmed.1005129

**Published:** 2026-06-04

**Authors:** Andrew S. C. Yuen, Kenneth K. C. Man

**Affiliations:** 1 Research Department of Practice and Policy, School of Pharmacy, University College London, London, United Kingdom; 2 Centre for Medicines Optimisation Research and Education, University College London Hospitals NHS Foundation Trust, London, United Kingdom

## Abstract

In this Perspective, Kenneth Man and Andrew Yuen highlight a recent PLOS Medicine study that presents new evidence on the safety of first-trimester NSAID use and congenital malformation risk, but discuss why interpreting findings across studies is challenging.

## Teaser

Pain and fever in pregnancy require treatment, but fetal safety concerns complicate analgesic choice. A recent *PLOS Medicine* study presents new evidence on the safety of first-trimester NSAID use and congenital malformation risk, but interpreting findings across studies is challenging.

Pain and fever are common in pregnancy, yet their treatment is complicated by concerns about fetal safety [1–3]. Paracetamol has long been regarded as the first-line option, but recent regulatory attention to possible neurodevelopmental risks has renewed uncertainty about analgesic use in pregnancy [4]. Non-steroidal anti-inflammatory drugs (NSAIDs), such as ibuprofen, diclofenac, and naproxen, offer alternative analgesic and antipyretic options, but the evidence on safety has been inconsistent, with some earlier studies raising concerns about birth defects [5,6]. Against this backdrop, a recent study in *PLOS Medicine* by Hasidim and colleagues [7] attempts to answer an important clinical question for pregnant women: Are recorded first-trimester NSAID dispensations associated with major congenital malformations?

The study’s headline finding is reassuring: first-trimester NSAID exposure was not associated with major congenital malformations overall, nor with any organ-specific category, including cardiovascular, musculoskeletal, central nervous system, gastrointestinal, or genitourinary malformations. Drug-specific analyses covering ibuprofen, diclofenac, naproxen, etodolac, and indomethacin returned similarly null results, although estimates for less frequently used agents were limited by small sample size. A dose–response analysis spanning short-term (1–7 defined daily doses) to long-term (>21 defined daily doses) exposure also showed no escalating risk signal.

Earlier studies that raised alarms were often small observational studies that compared NSAID-exposed early pregnancies against unexposed ones without adequately accounting for why the drug was prescribed, a problem epidemiologists call confounding by indication [5,6]. Hasidim and colleagues took several steps to address this. Firstly, they used directed acyclic graphs to guide covariate selection. They then applied propensity score matching so that retained NSAID-exposed pregnancies will have similar measured characteristics and probability of receiving NSAIDs when compared with unexposed pregnancies, allowing a fairer comparison between groups. In addition, they estimated risk ratios using G-computation, a causal inference framework that targets the average treatment effect rather than merely adjusting for observed differences. They also used dispensation data that captured a substantial proportion of prescribed and health-system-recorded over-the-counter NSAID use—including elective pregnancy terminations for fetal malformations that are often missed in birth-only cohorts—and followed children through the first year of life to identify malformations diagnosed after birth. These are improvements from some previous studies that alarmed clinicians in the past decade. Nevertheless, as the authors acknowledge, the findings are constrained by limitations of the underlying data: unrecorded private NSAID purchases, actual medication intake, and the absence of spontaneous abortions. The latter is particularly important because the evidence on early-pregnancy NSAID exposure and miscarriage remains mixed with some studies have reported increased risks, whereas others have reported elevated but statistically non-significant associations [8–10].

## The causal elephant in the room

The reassuring findings from this study come with an important caveat. The central methodological difficulty in perinatal pharmacoepidemiology is not merely whether you adjust for confounding, it is whether your adjustment strategy is temporally valid. First-trimester NSAID exposure in real clinical practice is not a clean, instantaneous event. Women take medications sporadically, sequentially, and often in combination. A woman may take ibuprofen for three days for fever, switch to paracetamol when her temperature settles, then return to an NSAID for musculoskeletal pain two weeks later. Throughout, her underlying condition is itself associated with adverse fetal outcomes; untreated fever in the first trimester has been linked to neural tube defects, congenital heart anomalies, and neurodevelopmental impairment [2].

**Fig 1:**
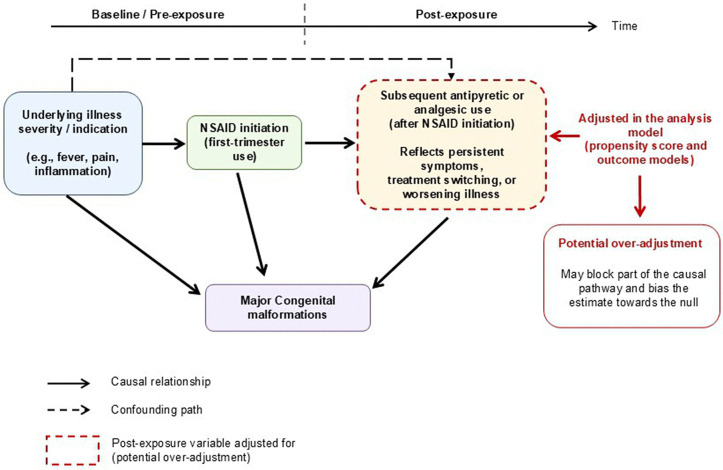
Directed acyclic graph illustrating the risk of over-adjustment when controlling for subsequent antipyretic or analgesic use after NSAID initiation in early pregnancy. Unmeasured factors other than underlying illness severity are not shown. NSAID, non-steroidal anti-inflammatory drug.

This creates a consequential problem. In this study [7], concurrent paracetamol and dipyrone use during the first trimester was included as a covariate in both the propensity score model and the outcome model, a reasonable attempt to account for unmeasured indication severity. When alternative analgesics are used after NSAID initiation, they may reflect evolving illness severity, persistent symptoms, or treatment switching rather than pre-exposure confounding. Conditioning on these downstream variables can introduce overadjustment, potentially moving estimates toward the null (Fig 1). The authors recognized this possibility and conducted a series of sensitivity analyses to examine it; the null findings remained largely unchanged.

This analytical rigor is reassuring, but it also illustrates the limitation of treating medication use as a single exposure. Analgesic use in pregnancy is often intermittent, sequential, and tied to evolving symptoms. Where data permit, alternative approaches such as marginal structural models or the parametric g-formula could help distinguish baseline indications, treatment switching, cumulative exposure, and gestational timing. As data infrastructure and causal methods mature, these can be applied in future perinatal pharmacoepidemiology research.

## Implications for clinical practice and future research

What should a GP or obstetrician take from this study? The findings support a cautious, rather than definitive, message: short-course NSAID use in the first trimester does not appear to substantially increase the risk of major congenital malformations. This matters because the underlying conditions carry their own documented hazards to the offspring. Clinical decisions should therefore weigh potential medication-related risks against well-documented harms of undertreated maternal illness, including fever-associated neural tube defects and the maternal-fetal consequences of unmanaged pain and inflammation.

Importantly, this study does not resolve the parallel uncertainty around paracetamol; clinicians are left to navigate an analgesic landscape in which neither first-line option offers unambiguous reassurance, and shared decision-making with patients about realistic trade-offs has become more important than ever. Absence of evidence for major congenital malformations should not be interpreted as evidence of safety across all pregnancy outcomes. The findings should not be extrapolated to later pregnancy, where NSAID-related fetal renal and ductal risks are well recognized, particularly after 20 weeks’ gestation [11]. Moreover, the broader evidence base is not consistent. An earlier South Korean nationwide cohort study published in *PLOS Medicine* reported modestly increased risks of congenital malformations associated with early-pregnancy NSAID exposure, as well as higher risks of low birth weight and oligohydramnios [12]. These discordant findings may partly reflect differences in exposure definition, comparator groups, and outcome ascertainment, while differences in population characteristics and healthcare systems provide additional context and underscore the need for further multinational studies.

## Conclusions

Hasidim and colleagues’ findings are clinically meaningful and reassuring, but not definitive, particularly given conflicting evidence from recent studies and the absence of data on outcomes beyond major congenital malformations. Future multinational studies should move beyond binary first-trimester exposure definitions and examine whether risks vary by drug class, including selective COX-2 inhibitors, dose, duration, gestational timing, indication, concomitant treatments, and broader offspring outcomes. For now, NSAIDs are not the teratogenic villains some feared, but neither are they unambiguously safe; the honest answer to a pregnant patient asking which analgesic to take is that the evidence is improving but still incomplete.

## Abbreviation:

NSAIDs: non-steroidal anti-inflammatory drugs
